# Automated segmentation of the fibula from CT imaging using two-stepped deep learning in 3D U-Net architectures

**DOI:** 10.1038/s41598-025-29130-y

**Published:** 2025-11-25

**Authors:** Jônatas de Souza Nascimento, Tobias Pankert, Florian Peters, Frank Hölzle, Ali Modabber, Mathias Wien, Stefan Raith

**Affiliations:** 1 Department of Oral and Maxillofacial Surgery, RWTH Aachen University Hospital, Aachen, Germany; 2 Institute for Imaging and Computer Vision, RWTH Aachen University, Aachen, Germany; 3 Inzipio GmbH, Aachen, Germany

**Keywords:** Anatomy, Computational biology and bioinformatics, Engineering, Health care, Mathematics and computing, Medical research

## Abstract

This study proposes a fully automatic segmentation of the fibula bone from CT images for application in pre-operative planning of reconstructive surgery. The objective is to make use of new developments in the image segmentation field to optimize and reduce the costs of patient-specific surgery planning. Two different approaches are proposed to perform the fibula bone segmentation, both based on a two-step segmentation method using a 3D-UNet architecture. To account for the symmetry of the left and right fibula bones, input images of the right fibula are mirrored to the left side. The accuracy of the trained models is measured using common evaluation metrics, together with specific metrics focused on facial reconstructive surgery. Both of the described approaches achieve high-accuracy results. For the best-trained model, an average Dice score of 0.95 and Average Surface Distances below 0.31 mm is measured on the test set in the region of interest for the surgery. Both approaches are robust segmentation techniques and permit data pre-processing for further application in the context of preoperative surgical planning of procedures for facial reconstruction with bony transplants.

## Introduction

In recent years, Convolutional Neural Networks (CNNs) have been used for different tasks in the medical field, including detecting tumors^1^, COVID-19 diagnosis^2^, and improving patient-specific individualization of treatments^3^. These exemplary applications show the enormous potential of using Deep Learning methods to improve different tasks in the medical field. There are, however, several applications in this domain for which the possibilities of using Artificial Intelligence-based techniques have not been explored yet. The planning of facial reconstructive surgery is an example where essential tasks could be potentially improved with such novel methods. Until now, few works on that topic have been published in literature, with only one publication known so far that deals with the segmentation of the iliac crest (i.e. a part of the pelvic bone) with the aim of use in reconstructive facial surgery^4^. There has been a remarkable increase in the use of computer tools to assist surgeons in the surgery planning procedure, which is leading to essential transformations in the field^5,6^. The use of Deep Learning techniques, commonly used in medical imaging, has remained scarcely studied with respect to an application in a surgical context. This can be seen in the facial reconstructive surgery planning context. Even though the use of 3D models of the donor bones during the planning procedures is shown to yield positive outcomes^7^, the usual process to acquire those 3D models is still highly manual, and time-consuming^8^, and could potentially be automated with Neural Network-based techniques. Even though fibular segmentation may appear less challenging compared to more complex anatomical structures, such as the mandible, an accurate representation of the fibular bone from CT scans is of paramount importance for a precise patient individual planning of reconstructive surgery. Although manual segmentation is feasible, it remains time-consuming and specifically subject to inter-operator variability, and thus bottleneck in an otherwise largely automated process routine of digital surgical planning. Thus, an automatic and hence repeatable and fast segmentation method, is highly valuable for clinical application.

While various fully automatic CNN-based approaches for the segmentation of different bones are described in literature^9–11^, segmentation of the fibula bone has not been investigated, despite its major significance as a donor bone for grafting procedures^12,13^.

In this study, we propose a fully automatic CNN-based approach to perform the segmentation of the fibula bone from CT imaging using a two-step, 3D-Unet approach. This two-step based approach using 3D U-Nets was chosen given its showed robustness and applicability in a variety of usage scenarios in medical image processing. Its application proved to be well-suited for the segmentation of other anatomical structures, including mandible^9^, the carpal bone^14^, and the pelvic bone^4^.

Thus, the contribution of automated segmentation of the fibular bone is a closing link to the process chain of a fully automated pipeline of surgical planning comprising mandibular segmentation^9^, graft design^15–18^ and finally the design of surgical guides^19^ to enable a high-quality digital workflow in mandibular reconstruction using fibula-free flaps.

### Virtual planning in reconstructive surgery

Preoperative planning is an essential part of complex surgical processes. Recently, there has been increasing research attention on this step^6,20^, recognizing that well-planned procedures positively impact the surgery as a whole^7^. This focus has guided the growth in the search for patient-specific planning solutions in the field of facial reconstructive surgery in recent years. Consequently, the use of computer-assisted tools in surgical preparation has gained special attention, particularly in mandibular reconstruction surgeries^21^. With the assistance of 3D modeling of the bones, a patient-specific cutting guide can be developed beforehand, along with osteosynthesis plates^22^. These advancements optimize the surgery in ways that were not possible employing just a freehand surgical approach.

Various researchers compared the mandibular reconstruction surgery when made with and without the assistance of virtual planning guides, where 3D models of the relevant bones are made based on CT images. Results showed significant improvements in operative time and the overall surgery together with better comfort to the surgeon^7^, better reliability^23^, and increased symmetry compared to freehand procedures^24^. Other studies also made systematic reviews of the impact of computer-assisted surgery in maxillary reconstruction based on available literature^6,20,21^. While there are relevant aspects of criticism on the heterogeneity of the evaluation metrics used to measure the success of the procedures^6^, they also highlight the advantages of computed assisted surgeries on the field^20,21^.

### Medical image segmentation

It is noted that, even though the use of virtual surgery planning has been showing great advantages in mandibular reconstruction surgery in general, the key limitation to this approach is the preparation of the 3D models to be used in the procedure. The first step of this process is to acquire three-dimensional image data of the anatomical regions of interest, typically with CT for bony structures, and then perform segmentation to annotate the bone to be modeled before the surgery. For instance, from the CT scan of the bottom region of the body, extract only the fibula that will be used as a donor bone for the surgery. The simplest and yet most commonly used segmentation method in the surgery planning context is threshold segmentation^8^. It consists of a selection of a threshold of intensity and asserting the value one for every pixel in the image above the intensity threshold, or zero otherwise^25^. The threshold can be selected either empirically or using a quantitative technique. Van Eijnatten et al.^8^ made an overview of segmentation techniques for additive manufacturing of anatomical models. Their analysis shows that most segmentation applications used either a direct global threshold or some variation of this approach. However, even though this method was proven to be capable of achieving favorable results with decent geometric accuracy, the method includes an extensive manual step. This is because the cited method has a series of inconsistencies, such as not taking CT artifacts and noise into account or intensity variations between different CT scanners^10^. So far, the only way around such inconsistencies when creating 3D models suitable for surgery planning procedures or similar tasks includes manual post-processing, which is usually expensive, time-consuming, and requires a skilled and trained expert. Even in the best cases, due to subjectivity or personal variance, the final results of threshold-based segmentation may vary significantly.

To overcome these problems of purely threshold-based segmentations, altas-based approaches showed remarkable performance, e.g. for femur segmentation^26^. Deep learning techniques are more recently widely studied in the context of medical image segmentation. Different studies proposed CNN-based techniques to segment bones from CT^27,28^ or MRI images^29,30^. Yosinski et al.^31^ showed that initializing weights of deep neural networks based on an already trained model improves the generalization performance of the model, even for non-related tasks. This feature is called Transfer Learning and was already used in the medical field, e.g., to train networks to identify COVID infection based on chest X-rays^32^, to classify brain tumor MRI images^33^, and also to classify endoscopic colonoscopy images^34^. Zhang et al. could show that even segmentation of different facial bones and corresponding landmark detection based on a heatmap approach is feasible^35^.

Focusing on facial reconstructive surgery planning, Pankert et al.^9^ developed a U-Net-based two-step segmentation approach to segment the mandibular bone. They conducted an extensive discussion on the advantages of using a two-step approach instead of only one U-Net and reached Dice scores of over 0.94 and Average Surface Distances lower than 0.36 mm. The present work intends to investigate the possibility of adapting this two-step segmentation method to the donor bones of facial reconstructive surgery. This way contributes to the current main issue of the planning procedure, which is the high costs and time consumption of the segmentation step.

## Materials and methods

### Dataset overview

The RWTH Aachen University Hospital provided CT scans of 89 patients for this study along with corresponding manual segmentations of the fibula bone that were used in real surgery planning scenarios. Institutional approval (EK 260/20) of the local ethics committee of RWTH Aachen University Hospital was obtained. All methods were carried out in accordance with relevant guidelines and regulations.

These segmentations are referred to as ground truth in the following. The age of the patients ranged from 11 to 80 years with a mean of 55.9 and a median of 59 years. 37 patients were male and 52 female. The dataset provided was not fully homogeneous and partially contained inconsistencies, so the data was carefully investigated for those flaws, and sets were excluded from the subsequent study in case they did not meet the inclusion criteria, that was defined as a complete manual segmentation with correct spatial alignment to the corresponding CT imaging data. Additionally, not all patients had both fibula bones segmented, but only one side. In these cases, the available side was chosen for subsequent work. The included datasets were subsequently used in the convolutional neural networks training that is detailed in further sections.

After the exclusion of the inconsistent cases, there were 13 data sets with only the right fibula, 26 data sets with only the left fibula, and 36 data sets with both fibulae segmented, summing up to 111 fibulae from 75 subjects. From these, 10 data sets with both fibulae segmented were held back as an independent test set, to evaluate the proposed segmentation pipelines. This dataset was deemed to be sufficiently large to produce satisfying results, as other studies with two-staged approaches showed to be applicable with smaller datasets, e.g. on carpal bones^14^ and in a preliminary study on training set sizes on the mandibular bone^9^. However, considering the wide range of ages and the inclusion of both sexes, the relatively small dataset size is a limitation of the study.

### Segmentation approach

In this work, we present two different methods to automatically segment the fibula bone from the CT data. The development of these two different approaches was done because of the dataset specificity that the bones of interest appear twice in the field of view. Both of these propositions are modifications of methods previously studied in literature. Wang et al., for instance, proposed a two-step segmentation framework to segment organs at risk of the head^36^. The method uses a two-step segmentation process that starts with a localization step that is applied to the whole field of view of the imaging data. The purpose of this initial step is to locate the region of interest around the anatomical structures of relevance in order to crop the field of view around this detected region and to allow for a subsequent detailed segmentation. Subsequently, a second step, that may be called a refinement step, is applied that can focus on the structures of choice and allows to segment these particular structures in detail with high precision by optimally using the available model capability. This method proved to be beneficial in a variety of different use cases, such as the segmentation of mandibular bones^9^, the pelvic bone^4^ or the wrist bone^14^, as shown in previous research. In this application, two U-nets are independently trained for each step of the task. For both steps, the same input and output resolution was used.

Therefore, the present study proposes to address the fibula bone segmentation on the available dataset. The baseline method is similar to the approaches described previously: Two U-Nets are trained to perform two different tasks. The purpose of the first U-Net is to segment the bone of interest from the full CT image, while the second is trained to segment the bone only from the bounding box where the bone is present.

#### Bilateral segmentation

The first proposed method consists of a two-step segmentation, where the first step is responsible for identifying both of the fibulae at the same time. Thus, the first step is trained on the whole CT image, down-sampled to the resolution $144 \times 144 \times 288$ voxels, and the output is the left and the right fibulae, which will be used for the second step of segmentation. To identify the left and the right fibulae, the output of the U-Net is converted into 3D surface objects using the marching cubes algorithm^37^. We assume that the two largest output meshes are the fibulae bones, while the rest are discarded. Based on their orientation in space, we label the fibulae meshes as left and right.

Similar to^36^ and^9^, the neural networks for the second step are trained only on the region of interest of the CT, for this application resampled to $80 \times 80 \times 960$, not on the whole data. However, since there are two fibulae, one on the right side and one on the left, two different U-Nets would need to be trained for this step. To circumvent this issue, we propose to mirror all right fibulae along the mid-plane of the field of view to the left side to train the second-step segmentation model on all fibulae simultaneously.

**Fig. 1:**
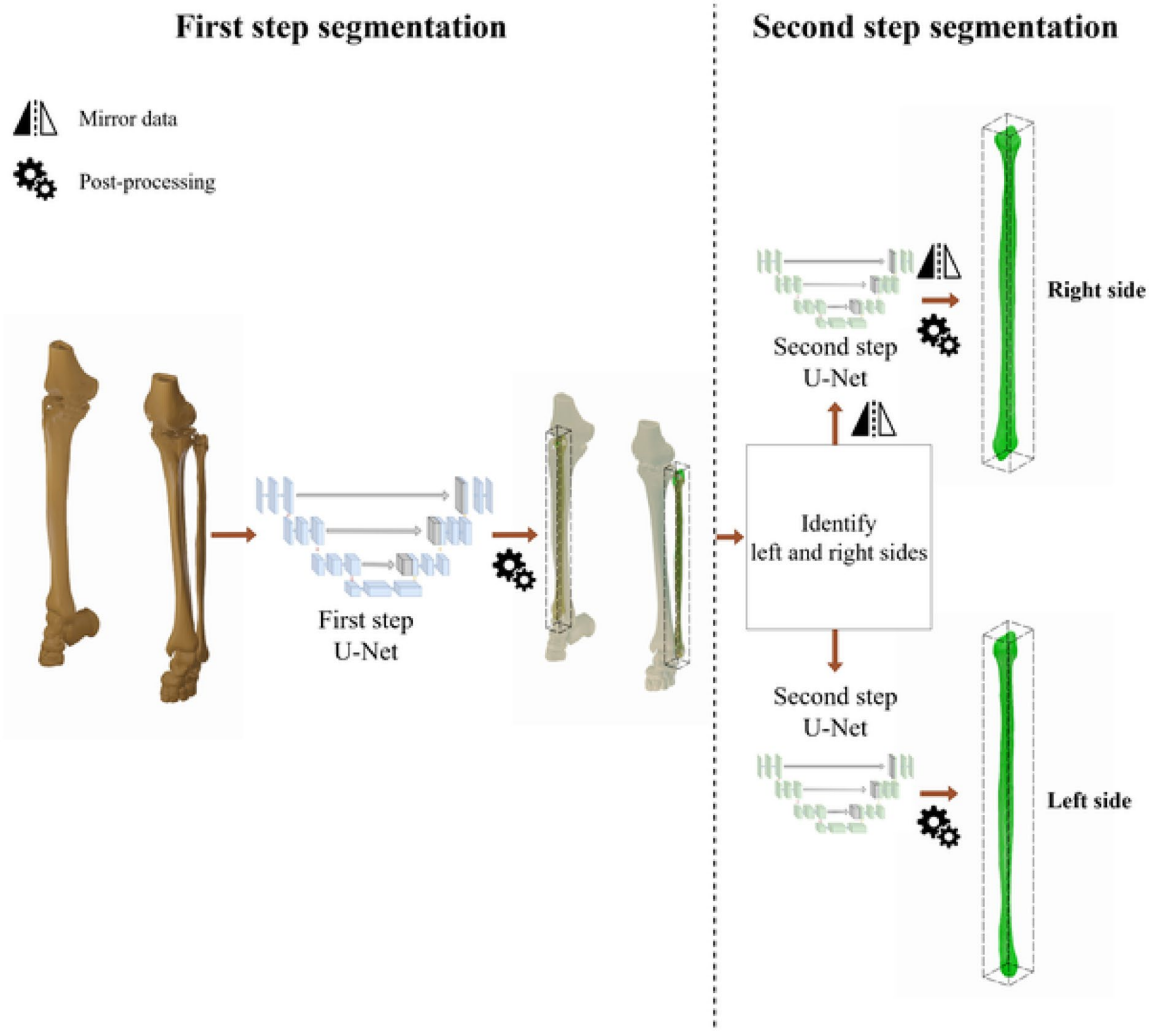
Bilateral segmentation approach overview: The first step takes the original CT data (shown in the diagram as threshold segmentations for visual clarity) and segments both fibula bones from the whole CT, then the second step segments each fibula bone from the CT cropped to the region of interest.

Hence, the entire pipeline consists of the following: Initially, the first step of the segmentation identifies both fibulae based on the full CT. Then the output results are post-processed and transformed into 3D meshes. Then the original CT is cropped to the bounding box of both right and left outputs, and the right fibula and cropped CT are mirrored. Lastly, the second-step model segments each of the fibulae from the cropped CT, then once more the output is post-processed and the right fibula output is re-mirrored. A diagram of this approach is shown in Fig. 1.

#### Unilateral segmentation

Even though the method presented above is solid, and produces remarkable results, as will be shown in the Results chapter, a relevant issue with this implementation is that for the first step model to be trained, ground truth data for both left and right fibula need to be available, otherwise, it can not be used for training. This is specifically problematic for the available dataset since there are only 36 patients with both fibulae segmented in the whole set, due to the nature of the data coming from real clinical cases for surgical planning. As the second step is trained for each fibula individually, all the data can be used in the training procedure, and no samples have to be discarded. For the first step of the bilateral approach, data from 39 patients were excluded, which is 52% of the complete set.

Therefore, we propose an additional approach for the first step, where potentially all the data can be used for training the U-Net models. The proposed method consists of using all the fibulae individually for training, similar to what is already done in the second step. Instead of segmenting both fibulae from the complete CT scan, the proposition is to split the CT in half along the mid-plane of the field of view, with each half containing only one fibula.This does not imply an exact alignment with the anatomical mid-sagittal plane, but in our examples, as all patients were lying centric enough on the bench with sufficient distance between their legs, thus this approach showed to be valid on the given data. Subsequently, the first step segmentation model is trained to segment the fibula based only on this half section of the image. To avoid training two different neural networks for the same task, the same mirroring technique used in the second step is also applied to this modified first step. Hence, the right side of the CT, which was cropped, is also mirrored along with the ground truth in this step and only one neural network is trained in this step to identify left fibulae from the left part of CTs. A different resolution is also necessary for this first step, since the region that will be input for the U-Net is no longer based on the full CT scan. To maintain the relative proportion, the proportion of voxels from the y-axis to the x-axis is doubled, and the proportion from the y-axis to the z-axis is kept the same. The chosen resolution therefore is $96 \times 192 \times 320$.

**Fig. 2:**
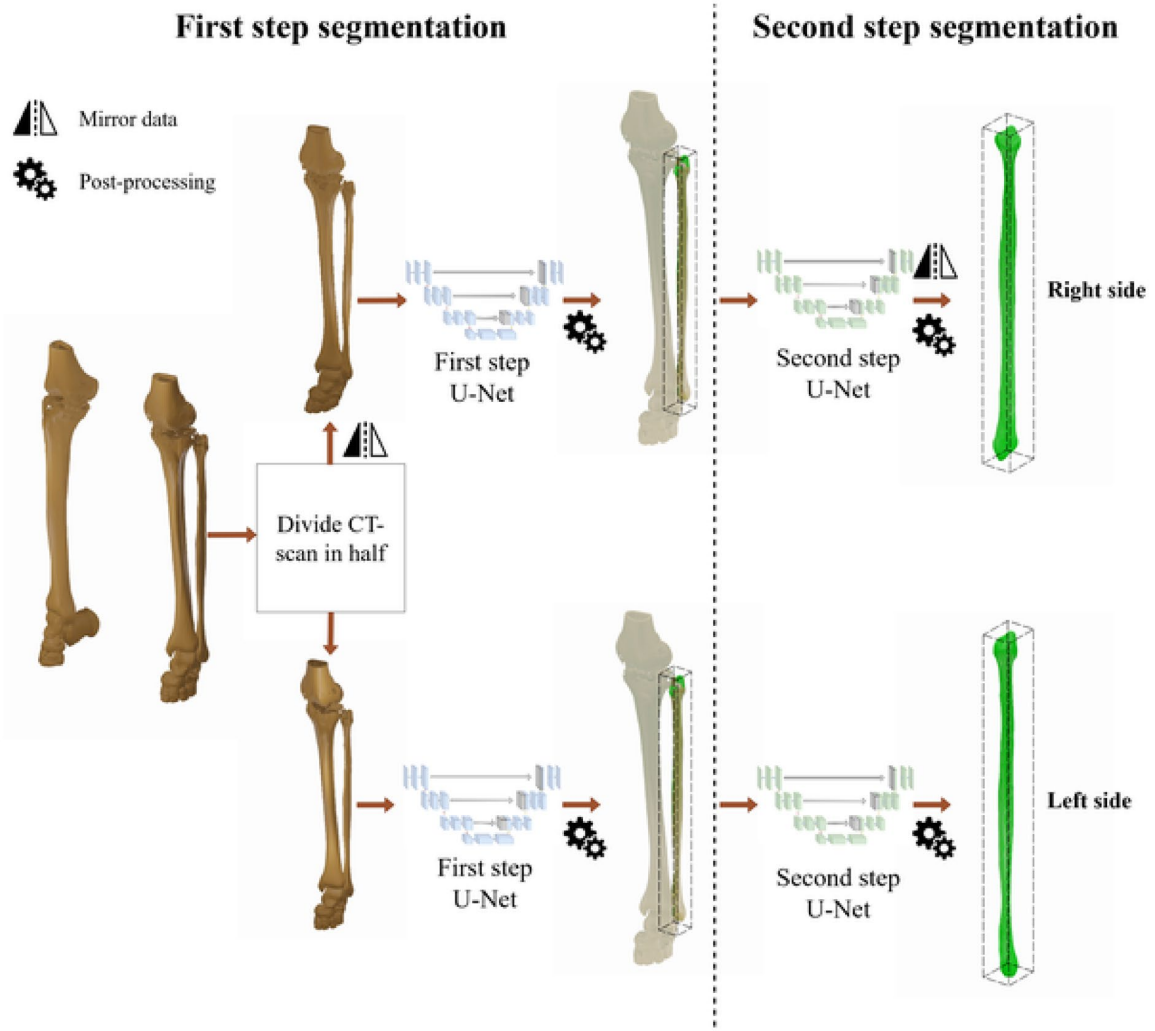
Unilateral segmentation approach overview: The CT dataset is divided into left and right sections (shown in the diagram as threshold segmentations for visual clarity), and then the first step segments each fibula bone from its respective side, and finally the second step segments each fibula bone from the CT cropped to the region of interest.

In conclusion, the method presented here involves first splitting the CT data in half on the lateral axis, then using the same first-step model to segment both the left and the mirrored right side of the fibulae. Finally, to apply the second step model to segment both fibulae from the CT images cropped only to the bounding box of the fibulae found in the first step. Figure 2 presents an overview of this approach.

#### Dataset distribution

After preprocessing of the raw imaging data and a technical check for data integrity, all available data was used for the training of the respective networks and divided into a training and validation set. For the training of the bilateral first step of this model, 22 could be made available, split with a 9 to 1 ratio into training set (n=20) and a validation set (n=2). The first step of the unilateral segmentation could use a database of 87 fibulae, where 80 could be used in training and 7 for validation. The second stage could rely on dataset of 85 fibulae, split into a dataset training (n=75) and validation (n=7).

### Transfer learning

As transfer learning has shown to be beneficial in related research^31–34^, both approaches proposed in this study use it. In this application, first, the second-step U-Net is trained from scratch, as this is expected to be a computationally easier task. Subsequently, transfer learning to the first step is used, as this showed superior results in preliminary work on other segmentation tasks. This allows for expanding the more detailed training results of the second step to the whole field of view of the CT, hence this approach is referred to as Expansion Transfer Learning (ETL)^14^.

### U-Net architecture

The U-Net architecture used for the experiments is a variation of the three-dimensional U-Net described in^38^. It has ten convolutional layers in the encoding path, with four max pooling layers in between. Following a common pattern seen in the literature^9,39^, it doubles the number of feature maps for every pooling step. The network uses $3 \times 3 \times 3$ receptive fields and $2 \times 2 \times 2$ max pooling layers. Furthermore, the decoding path consists of nine convolutional layers and five upsampling layers, which simply repeat the elements of the input tensor by an upsampling factor in every dimension. The upsampling factor used in the described architecture was $2 \times 2 \times 2$, which means the output of every upsampling layer is double its input for every dimension. An overview of the 3D U-net described above can be seen in Table 1. All networks described in this paper were trained using an Adam optimizer^40^ and a Dice loss. We did not implement data augmentation techniques in our presented methodology. While preliminary experiments were conducted with various augmentation approaches including spatial deformation, scaling, intensity variations, and noise addition, we did not observe significant improvements in model performance that would have justified their inclusion in our final implementation.

**Table 1:** 3D U-Net architecture.

	Type	Output feature maps
Encoding path	Input layer	1
	Conv	16
	Conv + Pool + Conv	32
	Conv + Pool + Conv	64
	Conv + Pool + Conv	128
	Conv + Pool + Conv	256
	Conv	512
Decoding path	Conv	512
	Conv + Upsampling + Conv	256
	Conv + Upsampling + Conv	128
	Conv + Upsampling + Conv	64
	Conv + Upsampling + Conv	32
	Conv	1
	Output Layer	1

No individual image normalization was used to standardize intensity values. However, the standard range of Hounsfield units (from -1024 to +3071 HU) was clipped and mapped to floating point numbers in the range from 0.0 to 1.0 for further processing in the neural network.

### Post-processing

Both proposed segmentation methods include a post-processing step of the model’s predictions. This stage occurs after all the neural networks outputs and transform the binary image output to the 3D model mesh, that is subsequently used in the surgical procedure. The first step of post-processing is a binary erosion of the data^41^ and is followed by the application of the Gaussian filter^42^ to add smoothness. Finally, the binary data is transformed to a mesh using the marching cubes algorithm on the predicted data^37^.

### Evaluation metrics

To evaluate the trained models in this study, we use three metrics commonly used in medical image analysis in similar applications. The first metric is the Dice Score (DS)^43^, an overlapping metric that compares the result segmentation and the ground truth. For a ground truth segmentation represented by the set of voxels *A* and a predicted segmentation represented by the set of voxels *B*, the Dice Score can be formalized according to Equation 1.

1 $$\begin{aligned} \mathscr{D}\mathscr{S} = 2 \cdot \frac{A \cap B}{ A + B } \end{aligned}$$

Additionally, we use two distance metrics, the Average Surface Distance (ASD)^44^, and the 95% Hausdorff Distance (95% HD)^45^ which measure the average, and the 95% highest distance between the ground truth and the model prediction respectively.

#### Restricted evaluation of transplantable bone

**Fig. 3:**
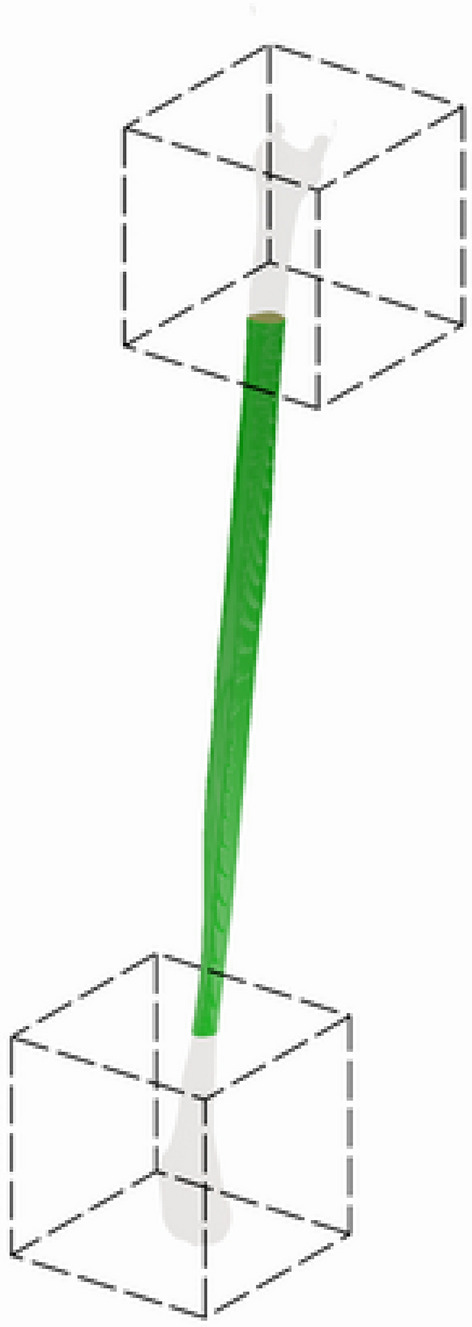
Fibula bone cropping example: the image shows the top and bottom 7cm being removed from the evaluation procedure.

During the facial reconstructive surgery with the use of the fibula-free flap, 6 cm or 7 cm from the top and bottom of the fibula are preserved to avoid knee or ankle instability^46–48^. Therefore, only the central part of the fibula is effectively used in the procedure and needs to be as well segmented as possible for the present application. Consequently, the proposition here is to evaluate with the same metrics as defined beforehand, removing the top and bottom 7 cm of the fibula bone. This removal can be seen in Fig. 3. This removal is done on both ground truth segmentation and the output of the proposed framework. Then the cropped meshes are again compared using Dice Score, Average Surface Distance, and 95% Hausdorff Distance. In this study, these metrics are additionally labeled as Region of Interest (ROI) metrics to differentiate from the standard implementations on the whole fibular bone.

## Results

The results section is split into a qualitative part and a quantitative part. Both refer to the final prediction, i.e. the output of the respective second U-Nets.

### Qualitative results

#### Bilateral segmentation approach

**Fig. 4:**
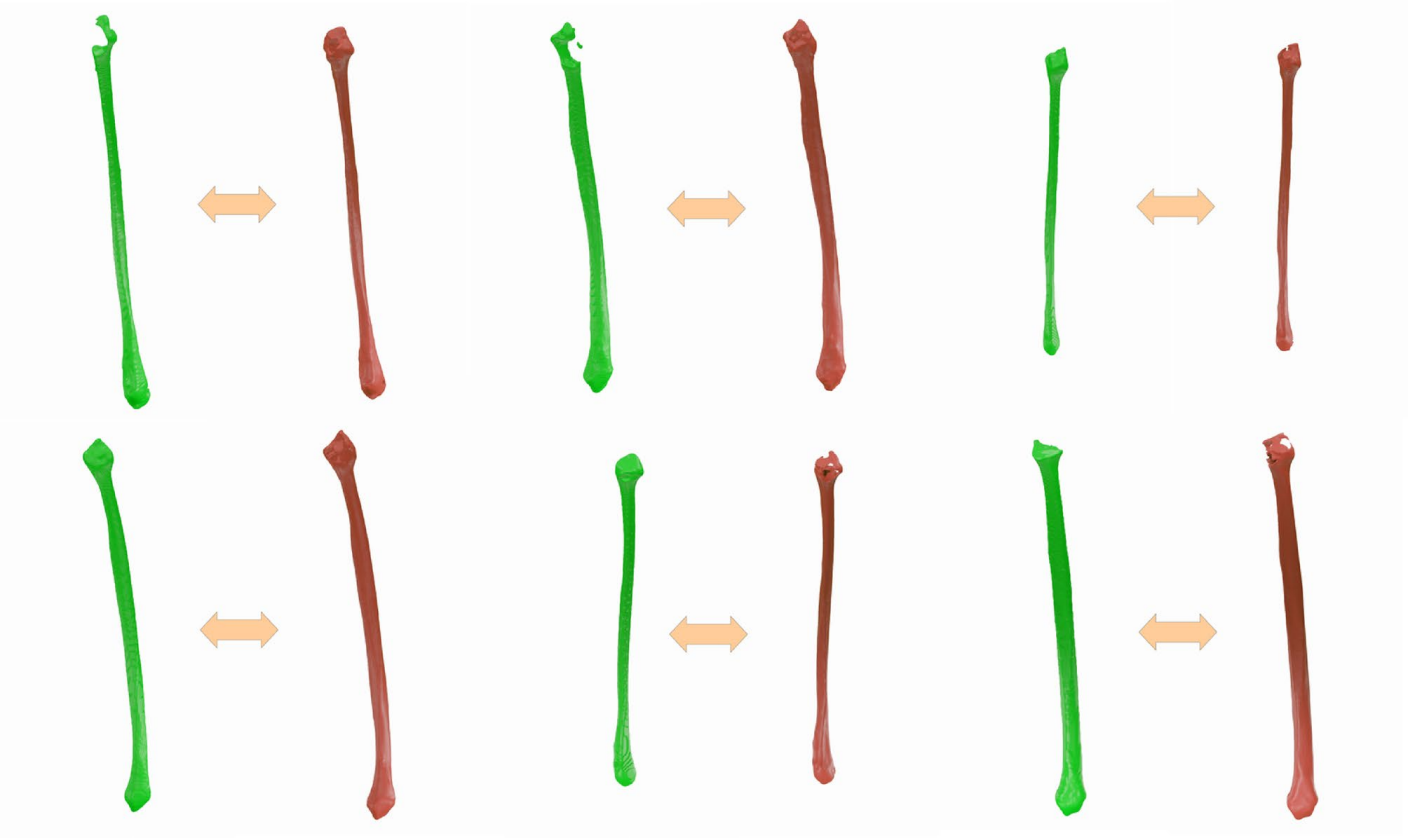
Bilateral Segmentation approach: Predictions (green) and ground truths (red) examples.

Figure 4 shows predictions from the trained models, each aside from its respective ground truth. The two neural networks combined succeed in identifying not just the fibula bone region and a fibula-like object, but the fibulae are visually close to the corresponding ground truth segmentation.

Visually, all the bottom edges and central regions look well segmented, whereas the top parts are not perfect for all cases. The first and second fibulae, especially, have flaws in the segmentation of its top regions, where the ground truth has it well segmented.

#### Unilateral segmentation approach

**Fig. 5:**
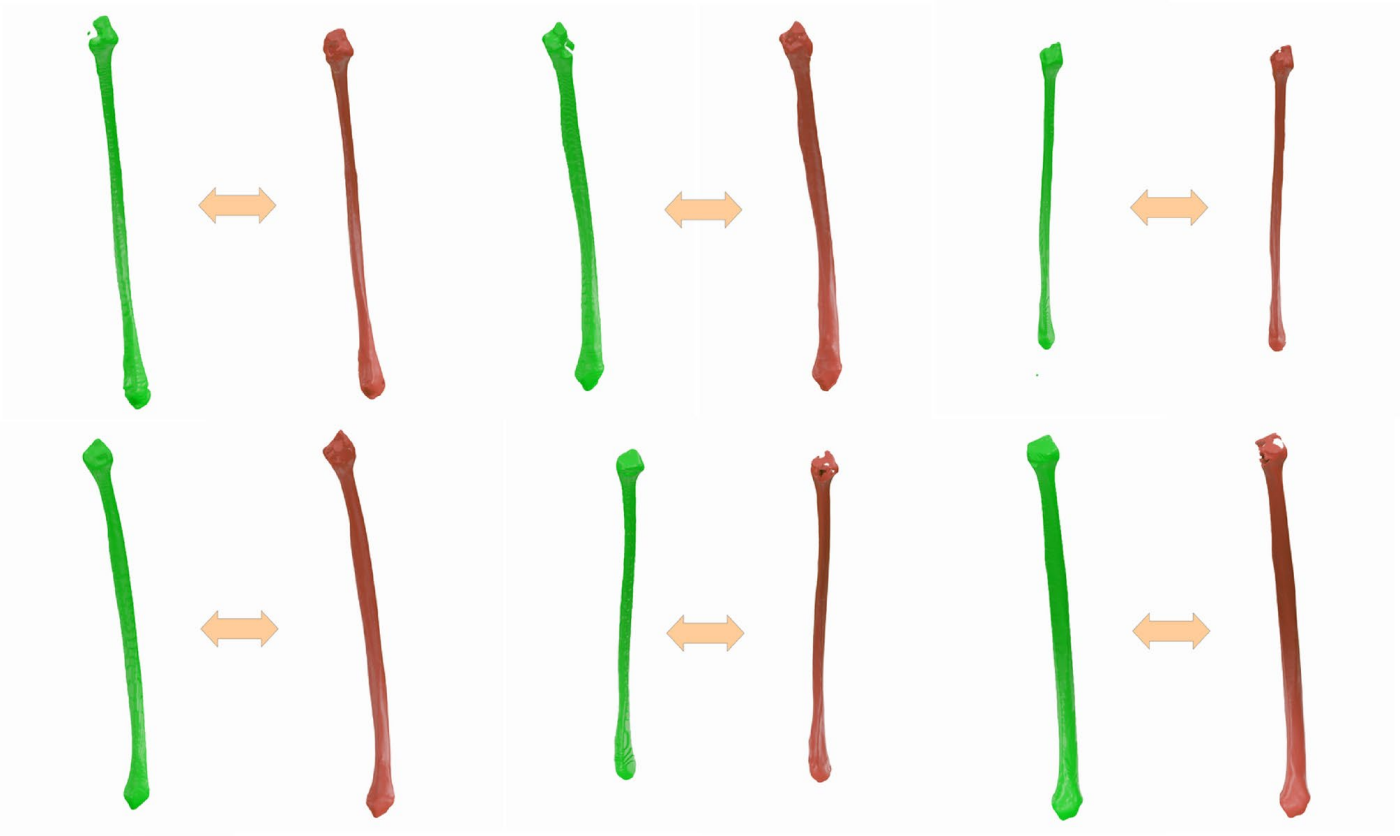
Unilateral segmentation approach: predictions (green) and ground truths (red) examples.

The predictions obtained using the unilateral segmentation approach can be seen in Fig. 5. These results were generated for the same fibulae as the ones in Fig. 4, therefore a direct comparison is possible.

**Fig. 6:**
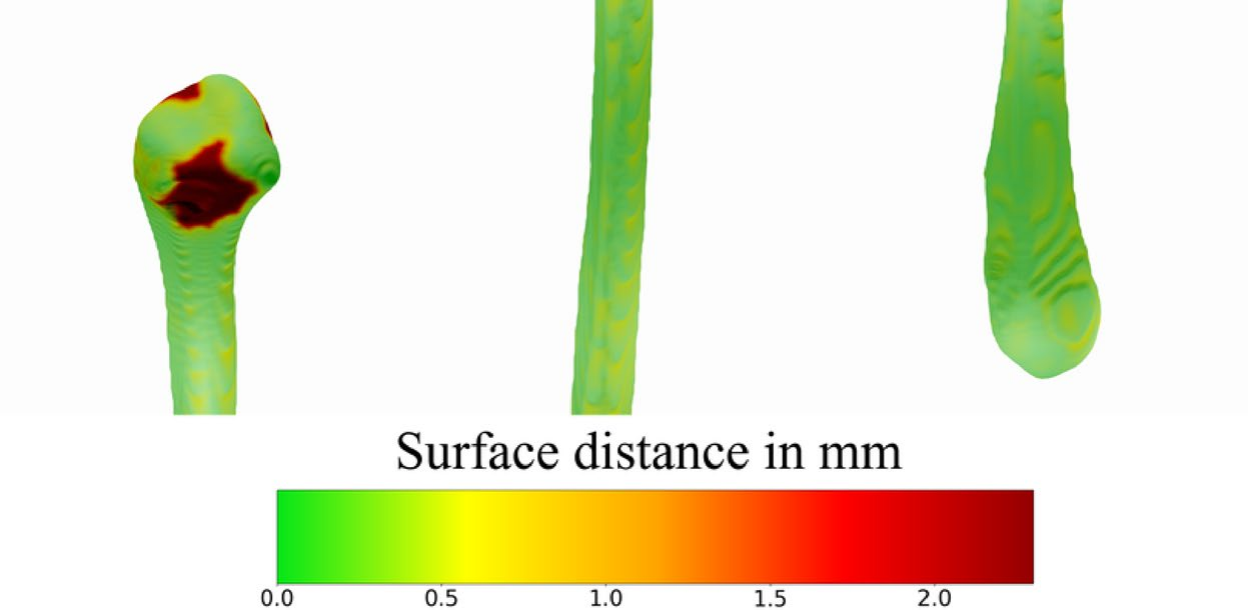
Detail view of a prediction in relation to ground truth data in color-coded 3D surface comparison. (left: proximal region of the fibula (head), middle: shaft of the fibula, right: lateral region of the fibula (ankle). The image shows an example result of the bilateral approach.

Visually, the predictions are closely aligned with the ground truths, especially on the central region, which is the one of biggest importance for facial reconstructive surgery, and also for the bottom region. The first and second fibulae show minor flaws in the top region segmentation, even though the segmentation looks relatively better than the ones achieved using the bilateral segmentation approach. Figure 6 displays a closer view of one example of prediction and highlights the poor results on the top region and the low deviation results on the central and bottom regions.

### Quantitative results

Table 2 displays the quantitative evaluation of the trained models on the test set.

**Table 2:** Evaluation of all models on the whole test set based on Dice Score (DS), Average Surface Distance (ASD) 95% Hausdorff Distance (HD) both on the whole fibular bone, and restricted to the Region of Interest (ROI).

Approach	DS	ASD	95% HD	ROI DS	ROI ASD	ROI 95% HD
Bilateral segmentation	$0.85 \pm 0.09$	$0.91 \pm 1.55$	$5.45 \pm 14.81$	$0.94 \pm 0.02$	$0.36 \pm 0.12$	$0.89 \pm 0.59$
**Unilateral segmentation**	$0.87 \pm 0.05$	$0.58 \pm 0.25$	$2.15 \pm 0.84$	$0.95 \pm 0.02$	$0.31 \pm 0.13$	$0.79 \pm 0.63$

The unilateral approach performs overall better in all metrics. When evaluating the metrics on the whole fibula, the achieved Dice Scores are 0.85 and 0.87 for the bilateral segmentation and the unilateral method respectively, while the Average Surface Distance and 95% Hausdorff Distance show an even bigger difference between the two approaches. It is also important to note the high standard deviation of the bilateral segmentation evaluation.

However, for the Region of Interest (ROI) metrics, the difference between the two approaches is less significant. Both methods achieve high DS values of 0.94 (bilateral approach) and 0.95 (unilateral approach), the ASD and 95% HD metrics are at 0.36 mm to 0.31 mm and 0.89 mm to 0.79 mm, respectively (Table 2).

**Fig. 7:**
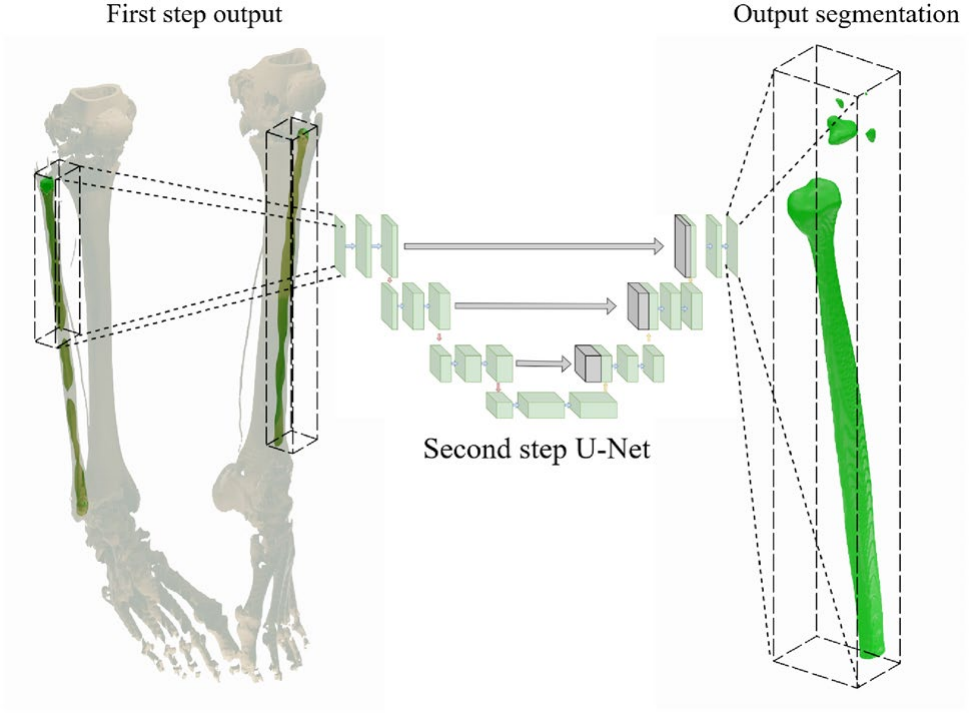
The low accuracy of the first-step segmentation in the bilateral approach leading to an incorrect bounding box for the second segmentation step. The outputs are displayed after conversion to mesh representations and before filtering out unconnected parts. Note: this is an exceptionally bad case and not the standard of our study.

The bilateral approach suffers from a robustness problem visible in the high standard deviations. For one of the test cases, the first step of the bilateral approach failed to predict one of the fibulae as a continuous bone. Due to filtering in the post-processing, the inferior part of the fibula was discarded, resulting in a too small field of view for the second segmentation step (Fig. 7).

**Fig. 8:**
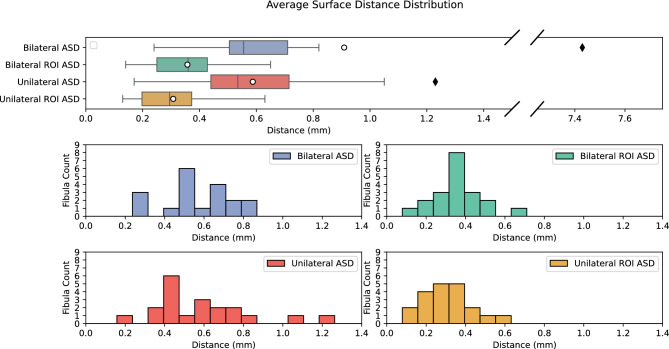
Distribution of the average surface distances (ASD) on the full bone or within the region of interest (ROI) for bi- and unilateral segmentations. For the histogram of the bilateral segmentations (in red), the outlier at 7.43 mm has been omitted.

Figure 8 shows the statistical distribution of the ASD evaluation on the test set for both the bi- and unilateral approaches. It is important to note that evaluating the same prediction only in the region of interest provides better results.

The unilateral approach solves the problem of high differences in the outliers. This variant achieves an average ASD and 95% HD value of 0.58 mm and 2.15 mm, respectively. Both standard deviations are drastically lower as well, which are 0.25 mm and 0.84 mm for the whole bone. Running the segmentations, including preprocessing of the DICOM datasets and all post-processing steps took on average 50 s for the bilateral and 58 s for the unilateral approach per dataset on a PC with an *Nvidia GeForce RTX 2080 Ti* and an *Intel Core i9-7900X*.

## Discussion

Even though the fibular bone is an important anatomy for diverse applications in the medical field, it is still not covered by research works on automatic segmentation of this bone, especially using AI techniques.

Though targeting a long bone of the lower extremity, the present work is particularly relevant for the field of maxillofacial reconstruction, where the fibula is commonly used as a donor site for autologous bone transplantation, and fast and reliable segmentation can significantly improve the preoperative workflow. Hence, the experiments conducted in this work are of high value to this clinical domain, as the proven applicability of this method in the given context demonstrates the efficiency for segmenting the desired parts of the fibula from CT images.

Therefore, the experiments conducted in this work are of high value to the field, since their remarkable results attest to the efficiency of the proposed methods in segmenting the desired bone from CT images. These results are especially good not just for the use case but have the potential to be used in different applications, such as fracture detection^49^^50^ or disease prediction^51^. Both methods proposed are modifications of the approach described in^9,36^. Overall, the results showed a high quality of the segmentation for both proposed approaches. When evaluating the predictions on the whole bone, the average of the Dice Score metric found for the two cases is over 0.84. Overall, the bilateral approach showed decreased robustness, as visible in the high standard deviations, due to the presence of outliers. The unilateral segmentation approach, by contrast, solves this problem and shows a significantly improved robustness.

This result is a strong indication of how the unilateral approach deals better with difficult cases than the bilateral segmentation approach. Even in CT scans for which the bilateral approach has difficulties in performing the first step segmentation, this method provides a segmentation that is sufficient to correctly detect the region of interest for the second step input. That the proximal part of the fibula shows weaker segmentation accuracy can be treated as irrelevant for the intended purpose of autologous transplantation. However, for potential application of this segmentation to other medical indications, the head of the fibula could deserve a more careful investigation, either by improving the ground truth segmentations in this particular region or by specific post-processing with shape-based refinement

Therefore, the visual analysis corroborates the statistics, which attest to an overall improvement of the metrics when using the unilateral approach when evaluating the whole bone. This is expected for two main reasons: The first one is directly related to the relative resolution of the down-sampling for the first step. Since the CT scan is divided in half for the unilateral approach, and the resolution used is kept proportionally, the relative down-sampling is lower for this case. Thus, for the bilateral method, more information is lost, which affects directly the training procedure. The second reason is related to the dataset availability. As described beforehand, not every patient in the data used for training has both fibulae segmented as a ground truth. This is a problem for the bilateral approach since it needs both bones to have ground truths to allow better training. Thus, the number of samples used for training in this method is 67.5% lower in comparison to the unilateral method. These factors combined explain how the split approach can deal better with the outliers, and have overall better results, even though the second step network is the same.

All these results together show that the unilateral approach is overall a better method to perform the fibula bone segmentation task than the bilateral approach, especially because of its higher robustness, and its remarkably better results for the metrics that evaluate the whole bone. Nevertheless, both the quantitative and qualitative analyses suggest that the main difference overall between the two methods is outside the region of interest. When evaluating both approaches only on the region of interest, the difference between them is shown to be significantly lower. Even the worst results of both metrics show Average Surface Distances below 1 mm in the fibula region used for the surgery. The other metrics show similar results, since the average of the Dice Score and 95% Hausdorff distance metrics are respectively 0.94 and below 1 mm for both approaches, and even the worst outliers are within a good range considering the task. These errors are all within the order of magnitude of the resolution of the underlying CT scans.

Thus, in summary, both two-step approaches showed to be applicable and yield remarkable accuracy, leveraging the full potential of the available hardware by applying a coarse segmentation in a first stage, followed by a detailed segmentation around the detected area of interest. In this approach, the advantages of using one common model architecture and training dataset could be combined with the gain in relative resolution in the focused second step.

For the application of the presented segmentation method in the context of surgical planning, the required accuracy depends on many different factors. Generally, the more accurate the segmentation the better for the surgical outcome. Accuracy is limited by several factors, including imaging, segmentation and manufacturing of parts created based on this data. Van den Broeck et al. report a mean error of 0.48 mm between CT scans and optical scans of the tibial bone diaphysis^52^, showing that the imaging already contains some errors in comparison to the real clinical application. Wallner et al. report an inter-observer Dice score of 0.94 between the manual segmentation of the mandible bone from CT imaging from two different human annotators^53^. Dice scores much higher than this might indicate an overfitting of the AI models to a specific human annotator instead of correctly generalizing to the segmentation problem. In a work by Kim et al., the manufacturing accuracy of surgical guides based on different manufacturing techniques was measured, showing mean errors ranging from 0.09 mm for SLA printing to 0.31 mm for Multijet printing (MJP)^54^.

These results are of high importance for the present work, since they show that even though the bilateral segmentation approach has problems regarding robustness when considering only the region that is considered in the facial reconstructive surgery, the results are outstanding for all cases. Even though there are no studies that apply a similar approach to perform the segmentation of fibular bones, it is possible to compare the achieved results to other bone segmentation from volumetric images. For instance, when performing the vertebral cortical segmentation from chest CT scans using a 3D U-Net-based approach,^55^ obtains Dice Scores of only 0.71, which is considerably lower compared to the results achieved in the present work.^56^ proposed a cascaded approach to segment condyles from CT scans, also using 3D U-Nets. The results achieved in this study were of high quality, achieving over 0.93 Dice Score values and Hausdorff Distances lower than 2.45 mm. Focusing on the reconstructive facial surgery planning application, Pankert et al. developed a fully automatic framework to segment the mandibular bone and obtained averages of 0.94, 0.35 mm, and 0.97 mm for the Dice Score, Average Surface Distance, and 95% Hausdorff Distance respectively, and attested its significance and possible applicability in surgical planning scenarios^9^. All the discussed comparisons have to be done carefully since even though the metrics are the same, the segmented bones and the available datasets are significantly different. However, literature results attest to what is a good segmentation in the medical image field. In fact, for the region of interest, the results obtained for this work are either better or at least similar for both presented methods.

The complete process of segmentation (including pre- and post-processing) took less than one minute on average on a workstation PC with contemporary hardware. Even though the fibular bone might be an easier task for manual segmentation than other anatomical structures, the time for manual segmentation is expected to be much longer. Though there is no published data available on the average times for manual segmentation of the fibula from CT scans, and comparisons with the segmentation of other anatomical structures are not directly applicable. Based on internal consultation with personnel experienced in manual segmentation, an estimated duration of 5 to 10 minutes per fibula was reported. It is important to note that this estimate refers to trained professionals with substantial experience; for less experienced users or those performing the task only occasionally, a significant learning curve can result in considerably longer times. While this working time is lower than for more complex anatomical structures or regions with extensive imaging artifacts, it still represents a relevant burden in an already packed clinical workflow. Moreover, with an automated segmentation using a CNN, any inter-observer differences may be omitted.

Thus, the AI approach presented in this work, enables the integration into a comprehensive process chain of surgical planning, which involves more cumbersome tasks, and profits significantly from an objective and reproducible acquisition of bony geometry from CT data. The segmented virtual representation of fibular bones can be directly integrated into computer-assisted planning workflows, such as virtual preoperative planning of surgical reconstruction of the mandible or midface bones in continuity defects, as is required e.g. after tumor removal, bone necrosis, or excessive trauma, where an autologous transplant from the fibular bone in a so-called fibula free flap proved its clinical value^6,7,20,21^. The geometric accuracy of the virtual surfaces of the digital bone models is essential for the planning of the transplant to match the defect dimensions and thus allow for optimal functional and aesthetic rehabilitation.

This work focuses on the surgical planning of fibula bone transplants in reconstructive surgery. A critical factor for clinical success is the accurate identification and integration of supporting blood vessels, as visible in CT angiography. While the present study concentrates on bone geometry, a natural extension of the automated segmentation approach would thus be the integration of vascular structures. Enabling automatic detection of relevant vessels would allow this information to directly inform clinical decision-making without requiring additional manual input. However, this task is considerably more challenging due to the high anatomical variability of vascular structures and would likely necessitate a different study design, potentially with access to larger and more diverse datasets.

It is important to note some limitations of the study. The used dataset was prepared to focus on a facial reconstructive surgery context, therefore flaws in unimportant regions for the surgery were ignored when modeling the segmentations. This directly affected the predictions achieved by the trained models. Furthermore, the low number of data available for training, especially on the bilateral segmentation approach, was probably one of the causes of the model’s lack of robustness for this method. Thus, we expect the overall results to improve significantly when more data is available for training. More data could also be generated by applying data augmentation to the existing datasets. Even though within the limitation of the preliminary studies conducted prior to the present work did not yield a discernible improvement in performance, a further ablation on augmentations techniques and settings could achieve an improvement of the training performance. In future work, we intend to apply similar methods to segment other donor bones used in the facial reconstructive surgery procedure. Furthermore, comparisons of our CNN-based approach to Atlas-based methods^26^ might yield relevant insights to the technical specifics of the different approaches in this particular field.

## Conclusion

This study presented two methods to automatically segment fibula bones from full-resolution CT scans. Both propositions are based on two-step segmentations, where the second step predicts the output based on the region of interest found by the first step. The bilateral approach’s first step does a coarse segmentation of both fibulae from a full-resolution CT at once, while the unilateral approach first splits the CT into two lateral halves to simplify the task by increasing the relative image resolution and the usable training data.

Both alternatives presented in this paper have the potential to be used in clinical surgical planning scenarios after integration into approved software as a medical device. The framework is capable of producing accurate segmentation of the fibula bone from the CT scans with a significant improvement in speed in comparison to established (semi-) manual segmentation techniques, and can be directly used as input data for the computer-assisted planning procedure.

Even eventual imprecision can be easily manually corrected before the procedure, which would still be a better and overall cheaper approach than the state-of-the-art threshold segmentation-based techniques.

## Data Availability

The raw medical imaging datasets analyzed during this study are not publicly available due to privacy concerns, as per the guidelines of the ethics committee, which permits access to the original data only for a specific group of named researchers. However, evaluation data and statistical analyses derived from it are available from the corresponding author upon reasonable request.

## Code availability

The code for evaluation of the geometry data is available as part of the supplementary material of this publication. The base implementation of the utilized 3D U-Net is available under https://github.com/smatzek/3DUnetCNN. Further details of the implementation and methodological description, as well as specific code parts used in this study for the training and inference pipelines, are available from the corresponding author upon reasonable request.
